# Relation of Vertebral Deformities to Bone Density, Structure, and Strength

**DOI:** 10.1002/jbmr.150

**Published:** 2010-06-08

**Authors:** L Joseph Melton, B Lawrence Riggs, Tony M Keaveny, Sara J Achenbach, David Kopperdahl, Jon J Camp, Peggy A Rouleau, Shreyasee Amin, Elizabeth J Atkinson, Richard A Robb, Terry M Therneau, Sundeep Khosla

**Affiliations:** 1 Division of Epidemiology, Department of Health Sciences Research, College of Medicine, Mayo Clinic Rochester, MN, USA; 2 Division of Biostatistics, Department of Health Sciences Research, College of Medicine, Mayo Clinic Rochester, MN, USA; 3 Division of Endocrinology, Metabolism and Nutrition, Department of Internal Medicine, College of Medicine, Mayo Clinic Rochester, MN, USA; 4 Division of Rheumatology, Department of Internal Medicine, College of Medicine, Mayo Clinic Rochester, MN, USA; 5 Biomedical Imaging Resource, Department of Radiology, College of Medicine, Mayo Clinic Rochester, MN, USA; 6 Division of Computed Tomography, Department of Radiology, College of Medicine, Mayo Clinic Rochester, MN, USA; 7 Departments of Mechanical Engineering and Bioengineering, University of California Berkeley, CA, USA; 8 O. N. Diagnostics Berkeley, CA, USA

**Keywords:** bone density, bone quality, finite-element analysis, QCT, vertebral fracture

## Abstract

Because they are not reliably discriminated by areal bone mineral density (aBMD) measurements, it is unclear whether minimal vertebral deformities represent early osteoporotic fractures. To address this, we compared 90 postmenopausal women with no deformity (controls) with 142 women with one or more semiquantitative grade 1 (mild) deformities and 51 women with any grade 2–3 (moderate/severe) deformities. aBMD was measured by dual-energy X-ray absorptiometry (DXA), lumbar spine volumetric bone mineral density (vBMD) and geometry by quantitative computed tomography (QCT), bone microstructure by high-resolution peripheral QCT at the radius (HRpQCT), and vertebral compressive strength and load-to-strength ratio by finite-element analysis (FEA) of lumbar spine QCT images. Compared with controls, women with grade 1 deformities had significantly worse values for many bone density, structure, and strength parameters, although deficits all were much worse for the women with grade 2–3 deformities. Likewise, these skeletal parameters were more strongly associated with moderate to severe than with mild deformities by age-adjusted logistic regression. Nonetheless, grade 1 vertebral deformities were significantly associated with four of the five main variable categories assessed: bone density (lumbar spine vBMD), bone geometry (vertebral apparent cortical thickness), bone strength (overall vertebral compressive strength by FEA), and load-to-strength ratio (45-degree forward bending ÷ vertebral compressive strength). Thus significantly impaired bone density, structure, and strength compared with controls indicate that many grade 1 deformities do represent early osteoporotic fractures, with corresponding implications for clinical decision making. © 2010 American Society for Bone and Mineral Research.

## Introduction

Impaired bone quality, including architectural damage to trabecular bone, as assessed at the radius(1) and iliac crest,(2) increases with vertebral fracture severity, and there is a positive association between greater severity of vertebral deformities at baseline and a higher incidence of new vertebral fractures.(3) Indeed, we showed that the overall 2.8-fold risk of progression associated with any morphometrically defined vertebral deformity resulted largely from inclusion of the severe deformities; considered separately, severe deformities were associated with a 3.8-fold relative risk of progression compared with the nonsignificant 1.5-fold increase seen for mild deformities alone.(4) This raises the possibility that some mild (grade 1) deformities could represent measurement artifacts or other variants in vertebral body shape (ie, false-positive results). In a preliminary study comparing 40 postmenopausal women with vertebral fractures with 40 control women of similar age, we found little difference in areal bone mineral density (aBMD),(5) but the overlap may have resulted partly from misclassification of some mild deformities as fractures. If a substantial proportion of minimal deformities actually represents early osteoporosis, however, women with grade 1 deformities should differ significantly from age-matched women without deformities using more sensitive and physiologic measurements. To test the null hypothesis of no difference between controls and those with grade 1 deformities, we extended our preliminary study to include a larger number of postmenopausal women and complemented dual-energy X-ray absorptiometry (DXA) measurements with assessments of bone microstructure by high-resolution peripheral QCT (HRpQCT) at the radius and, more important, with direct estimates of vertebral body strength by finite-element analysis (FEA) of lumbar spine QCT images. We also compared control women and subjects with grade 1 deformities with postmenopausal women who had moderate to severe (grade 2–3) vertebral deformities. Although one recent study showed that assessments made at the radius better predicted severe than mild vertebral deformities,(1) we evaluated bone density and strength assessments made directly from the lumbar spine.

## Methods

### Study subjects

Following approval by Mayo Clinic's Institutional Review Board, we recruited community women 50 years of age and older who had a clinically diagnosed vertebral fracture within the past 5 years, augmented by women from a population-based study cohort who had vertebral fractures found on QCT lateral localizer (scout) images of T_3_ through L_5_ (ie, digital radiographs). These were compared with controls with no vertebral fracture who were recruited from the same age-stratified random sample of Olmsted County, MN, women.(6) Altogether, 283 of 368 eligible subjects (77%) participated. Women with vertebral fractures owing to severe trauma (eg, automobile accidents or falls from greater than standing height) or to a specific pathologic process were excluded, as was anyone who had undergone vertebroplasty or intermittent parathyroid hormone (PTH) therapy. Patients who had been treated with antiresorptive drugs [ie, bisphosphonates, hormone therapy, or selective estrogen receptor modulators (SERMs)] were included, however, because these agents do not appear to greatly alter bone structure.(7) All subjects provided written informed consent prior to participation in the study.

### Fracture ascertainment

Thoracic and lumbar vertebral body fractures were assessed from the QCT lateral localizer images, which have no projection distortion and a nominal resolution of 0.5 mm, by the study radiologist (PAR) according to the semiquantitative method.(8) Deformities were classified as mild (grade 1) or moderate to severe (grade 2–3).

### Bone density and structure measurements

Lumbar spine (LS), femoral neck (FN), and radius aBMD measurements were made by DXA using the Lunar Prodigy System (GE Healthcare, Madison, WI, USA), although 35 women had spine region aBMD assessed from a total-body scan on the same device; spine region scans are equivalent to dedicated LS DXA measurements in women, with *r* ^2^ = 0.84 and an error in predicting LS aBMD of 6.5%.(9) DXA spine scans were evaluated according to International Society of Clinical Densitometry (ISCD) criteria (http://www.iscd.org/visitors/positions/OPReferences.cfm). Thus vertebrae with deformities were deleted and the mean L_1_-L_4_ aBMD value recalculated from the remaining vertebrae. Osteoporosis was defined by World Health Organization (WHO) criteria using *T*-scores from the Lunar device.

FN and LS volumetric BMD (vBMD) and geometry were assessed by single-energy QCT using three different scanners over the course of the study: a 4-channel multidetector-row scanner (LightSpeed QX/i) and comparable 8-channel system (LightSpeed Ultra), both from General Electric Medical Systems (Waukesha, WI, USA), and a 64-channel system (Somatom Sensation 64) from Siemens Healthcare (Forchheim, Germany). Dimensions and scanner geometry were identical, and the same image-acquisition parameters were maintained. In addition, there was a change in external calibration standards from the Mindways Model 2 Liquid Calibration Phantom to the Mindways Model 3 Solid Calibration Phantom (Mindways Software, Inc., Austin, TX, USA). Crossover between the first two scanners and the two standards has been reviewed in detail,(10) and a similar method was used for crossover between the second and third scanners; conversion accuracy was confirmed using the European Spine Phantom (QRM GmbH, Möhrendorf, Germany). Briefly, the spine phantom was scanned on all three QCT devices, and we adjusted the values so that the overall vBMD was consistent between machines. Additionally, individual parameters (vBMD, bone area, etc.) were assessed for their relationship with age, and if appropriate, a further additive adjustment was made to ensure that measurements made for a given age, on average, were consistent between machines, as also described previously.(10) In addition to total vertebral vBMD, we also measured trabecular vBMD in the central 70% of the midportion of the vertebral bodies. A number of bone macrostructure measurements were made at midvertebral height, including total cross-sectional area, moment of inertia, section modulus, and “apparent” cortical thickness, recognizing both that this is not true cortical bone and that thickness of the cortical shell is overestimated in vertebrae owing to volume-averaging artifacts.(11)

In lieu of detailed trabecular microstructure data for the spine, we evaluated the nondominant wrist by HRpQCT (XtremeCT, Scanco Medical AG, Brüttisellen, Switzerland). As described elsewhere,(12) distal radius trabecular bone volume/total volume fraction (BV/TV) was derived from trabecular vBMD. A thickness-independent structure extraction was used to identify 3D ridges (centers of the trabeculae), and trabecular number (Tb.N) then was taken as the inverse of the mean spacing of the ridges. Analogous with standard histomorphometry, trabecular thickness (Tb.Th) was calculated as BV/TV ÷ Tb.N and trabecular spacing (Tb.Sp) as (1 – BV/TV) ÷ Tb.N. Tb.Sp.SD, the standard deviation of Tb.Sp, is a measure of trabecular variation. Validation studies show excellent correlation (*r* ≥ 0.96) of these parameters with “gold standard” ex vivo micro–computed tomography (µCT).(13) Trabecular architectural disruption also was assessed by connectivity density (Conn.D), whereas the structure model index (SMI) indicated whether trabeculae were more platelike (lower values) or more rodlike (higher values). The distal radius cortex was segmented from the grayscale image with a Gaussian filter and threshold.(12) Cortical vBMD and area were measured directly, and the periosteal circumference was calculated from the contour; cortical thickness (Ct.Th) then was calculated as area ÷ circumference. Excellent correlation (*r* = 0.98) also has been shown with Ct.Th measurements by µCT.(14)

### Estimation of vertebral strength characteristics

To estimate vertebral body strength, voxel-based FEA was carried out on the L_3_ vertebral body for each subject using custom software (O. N. Diagnostics, Berkeley, CA, USA). If L_3_ were fractured, L_2_ was analyzed (10 subjects) or L_1_ (1 subject). Seven women could not be evaluated (5 owing to image artifacts and 2 because of severe deformities). As described in detail elsewhere,(5) each vertebral image (less posterior elements) was rotated into a standard coordinate system and converted into a 1 × 1 × 1 mm^3^ mesh of 8-node finite elements, with the vBMD of each element used to estimate anisotropic material properties.(15) With endplates covered by a virtual layer of polymethyl methacrylate, each bone was virtually compressed to failure using nonlinear FEA, with overall compressive strength computed as the total reaction force generated at an imposed 2% deformation; this technique provides excellent measures of whole-bone strength.(15) To compute other variables, the simulation was rerun on altered models.(16) For example, intravertebral bone density variation was removed by applying the average vertebra-specific vBMD uniformly across all voxels of the finite-element mesh and computing the resulting “homogenized density” strength. Likewise, trabecular strength was estimated by removing the outer 2 mm of bone from the model and recomputing strength for the remaining trabecular compartment. The difference between whole-vertebra and trabecular strengths represents the strength associated with the outer 2 mm of “cortical” bone (thin cortical shell and adjacent bone). We also determined a ratio of bone strength to the average vBMD for each subject. Finally, to assess the response to anteroposterior (AP) bending loads, a pure bending rotation of 1 degree was applied to the top surface of the vertebra using linearly elastic analysis.(17)

### Load-to-strength ratio

The load-to-strength ratio ϕ compares the load a structure carries relative to its estimated strength so that higher values indicate an increased risk of structural failure. Theoretically, a fracture is predicted to occur when ϕ ≥ 1,(18) although the absolute fracture threshold is difficult to define in practice. Following a method similar to that used in earlier studies,(5) (19) ϕ was computed as the ratio of estimated compressive loads acting on the analyzed vertebra to the FEA-derived estimate of the overall compressive strength of that vertebra. For these calculations, the compressive load was estimated for four different cases: (1) upright standing, (2) bending forward 45 degrees at the waist with no weight in the hands, (3) bending forward at 90 degrees, and (4) bending forward at 90 degrees while holding 10 kg. Body segment weights and lengths were taken as percents of subject-specific total body weight and height, assuming that only the erector spinae actively exert force to counter the bending developed by forward flexion and act about the vertebra with a 5.48-cm moment arm. We also assumed that the posterior elements did not support load, and we considered only the compressive component of the reaction force on the vertebral body.

### Statistical analysis

Analyses were performed using SAS (SAS Institute, Inc., Cary, NC, USA) and R Version 2.7.0 (R Foundation for Statistical Computing, Vienna, Austria). Each bone variable was age-standardized by fitting a linear regression model using all subjects, extracting the residuals, and then adding to that the overall mean, that is, presenting the variables as if they were all measured on 70-year-old women. These age-adjusted bone variables were summarized using means and standard deviations.

The overall age-adjusted relative risk of fracture was estimated by odds ratios (ORs) obtained from logistic regression models, where the dependent variable was case status weighted according to the likelihood that each subject actually had a vertebral fracture. Thus, instead of forcing subjects into control (0) or case (1) groups, this allowed for an “equivocal” group (ie, the grade 1 deformities) whose case status was assigned an intermediate value of 0.25 (see details below). Logistic regression was run using this three-level endpoint to measure how well the independent variables predicted case status (0, 0.25, or 1). Traditional logistic regression models also were run comparing only the definite controls (no deformity) and definite cases (grade 2–3 deformity), the definite controls and equivocal cases (grade 1 deformity), or the equivocal cases and definite cases. The most significant independent predictor of fracture risk was assessed within each of five main variable categories—bone density, bone geometry, bone microstructure, bone strength (all per SD decrease), and ϕ (per SD increase).

Weighting for the intermediate fracture group (grade 1) was estimated using data from an earlier age-stratified population sample of 512 postmenopausal women who had serial radiographs for up to 12 years.(4) In that group, we assessed the relative risk of a subsequent vertebral fracture based on a quantitative morphometry categorization at baseline—no baseline fracture, one or more mild deformities (equivocal fracture status) or one or more severe deformities (definite fracture status). The relative risk of progression, defined by the 20% change in vertebral height criterion,(20) for the mild deformity group divided by the severe deformity group (ie, 0.25) was used to estimate the weighting for grade 1 (equivocal) deformities in our study cohort.

As an additional expression of fracture discrimination, the area under a receiver operating characteristic curve (*AUC*) was assessed by the probability of concordance (c-index), also obtained from the logistic regression models.(21) Comparisons of the *AUC*s were based on predictive values from the logistic models.(22)

## Results

Ninety postmenopausal women with no vertebral fracture (controls) were compared with 193 fracture patients who included 142 women with isolated (*n* = 40) or multiple (*n* = 102) grade 1 (ie, mild) deformities and 51 women with at least one grade 2 (*n* = 32) or grade 3 (*n* = 19) deformities (ie, moderate to severe) by the semiquantitative method; all but 10 of the latter group had multiple deformities (2 to 9 each). The women with severe deformities (76.2 ± 10.6 years) were significantly older (*p* < .001) than both the control women (66.2 ± 8.9 years) and the women with mild deformities (69.3 ± 10.2); the latter two groups also differed in age (*p* < .05). Reflecting the ethnic composition of the female population aged 50 years and over in the community (96% white in 2000), 98% of the subjects were white. Sixty-seven of the fracture patients had been diagnosed clinically with a vertebral fracture on average 3 years previously, whereas the remaining 126 had vertebral fractures found incidentally; clinical diagnoses had been made for 82% of the grade 2–3 patients but for only 18% of the women with a grade 1 deformity. Altogether, 44 women had fractures involving both the thoracic and lumbar spine, whereas 131 had only thoracic deformities and 18 had only lumbar deformities. Those with only grade 1 deformities were more likely than the others to have fractures confined to the thoracic spine (77% versus 41%), whereas women with at last one grade 2–3 deformity were more likely than the others to have fractures involving both thoracic and lumbar sites (47% versus 14%). In addition, more women with moderate to severe deformities had a prior history of a distal forearm or hip fracture (43%) than those with only mild vertebral deformities (26%); by design, none of the control women had experienced a prior osteoporotic fracture. Likewise, the prevalence of osteoporosis at the FN or LS varied from 32% among the women with a severe deformity to 13% in those with only mild deformities and 6% among the control women. Thirty-six percent of the patients (30% of women with only grade 1 deformities and 51% of those with any grade 2–3 deformity), compared with 31% of controls, were being treated with an antiresorptive agent at the time of study (mostly bisphosphonates in patients and estrogens in controls).

Patient height was lower among the women with any grade 2–3 deformity compared with controls (158 ± 7 versus 161 ± 6 cm, *p* < .05), but the height reduction in those with only grade 1 deformities (160 ± 6 cm) was not significant. More generally, the women with moderate to severe vertebral deformities had significantly worse values for almost every bone density, structure, and strength parameter than did the women with no deformities, even after adjusting for age, and they often differed from the women with grade 1 deformities alone (Table 1). However, the women with grade 1 deformities frequently had worse values than the women with no deformity, thus occupying an intermediate position between the women with no deformities and those with moderate to severe deformities.

**Table 1:** Age-Adjusted Comparison of Postmenopausal Women With and Without Specific Vertebral Deformities (Semiquantitative Assessment) With Respect to Five Predictor Variable Categories and Percentage Differences (Δ%) Relative to Women With No Deformity

Variable (units)	No deformity (*n* = 90)	Mild (grade 1) deformity only (*n* = 142)	Δ%	Any severe (grade 2–3) deformity (*n* = 51)	Δ%
Bone density					
Lumbar spine (LS) aBMD (g/cm^2^)	1.12 ± 0.16	1.09 ± 0.18†	−3	1.04 ± 0.17**	−7
Femoral neck aBMD (g/cm^2^)	0.88 ± 0.15	0.84 ± 0.12†	−5	0.79 ± 0.09***	−10
Total radius aBMD (g/cm^2^)	0.61 ± 0.08	0.59 ± 0.09††	−3	0.55 ± 0.10**	−10
LS total vBMD (mg/cm^3^)	185 ± 38	170 ± 35**,†††	−8	149 ± 28***	−19
LS trabecular vBMD (mg/cm^3^)	146 ± 32	133 ± 29**,†††	−8	115 ± 21***	−21
Distal radius trabecular vBMD (mg/cm^3^)	139 ± 42	132 ± 41††	−5	108 ± 42***	22
Distal radius cortical vBMD (mg/cm^3^)	870 ± 65	861 ± 67	−1	845 ± 82	−3
Bone geometry (lumbar spine)					
Cross-sectional area (cm^2^)	10.3 ± 1.1	10.5 ± 1.3	2	10.7 ± 1.5	4
Endocortical area (cm^2^)	8.58 ± 1.05	8.85 ± 1.24	3	9.08 ± 1.29*	6
Cortical area (cm^2^)	1.76 ± 0.24	1.71 ± 0.25	−3	1.65 ± 0.28*	−6
Apparent cortical thickness (mm)	1.68 ± 0.26	1.58 ± 0.24**,†	−6	1.48 ± 0.26***	−12
Bone microstructure (distal radius)					
Trabecular bone volume/total volume (%)	0.12 ± 0.04	0.11 ± 0.03††	−8	0.09 ± 0.03***	−25
Trabecular number (1/mm)	1.64 ± 0.38	1.55 ± 0.39†	−5	1.38 ± 0.43***	−16
Trabecular thickness (µm)	70.0 ± 11.2	70.5 ± 11.4††	1	64.5 ± 10.4*	−8
Trabecular separation (Tb.Sp, µm)	605 ± 308	638 ± 306†	5	770 ± 403**	27
Tb.Sp distribution (µm)	301 ± 231	346 ± 302†	15	453 ± 389**	51
Connectivity density (1/mm^3^)	3.06 ± 1.07	2.89 ± 1.07††	−6	2.36 ± 1.00***	−23
Structure model index	2.32 ± 0.39	2.35 ± 0.37††	1	2.57 ± 0.28***	11
Cortical thickness (mm)	0.82 ± 0.21	0.78 ± 0.20	−5	0.71 ± 0.21**	−13
Bone strength (lumbar spine)					
Overall compressive strength (N)	5528 ± 1898	4952 ± 1565*,††	−10	4089 ± 1344***	−26
“Cortical” strength (N)	2976 ± 717	2796 ± 711††	−6	2486 ± 702***	−16
Trabecular strength (N)	2852 ± 1289	2493 ± 956*,††	−13	1931 ± 675***	−32
“Homogenized” trabecular strength (N)	3912 ± 1609	3466 ± 1241*,††	−11	2853 ± 1072***	−27
Strength per unit density (N · cm^3^/mg)	31.5 ± 4.9	30.9 ± 4.7†††	−2	28.2 ± 5.2***	−10
AP bending stiffness (kNm/rad)	2.39 ± 0.85	2.22 ± 0.78†	−7	1.94 ± 0.73**	−19
Load to strength (ϕ, lumbar spine)					
Upright standing	0.08 ± 0.03	0.09 ± 0.03*,†††	12	0.12 ± 0.05***	50
45-Degree forward flexion	0.35 ± 0.12	0.39 ± 0.14*,†††	11	0.50 ± 0.22***	43
90-Degree forward flexion	0.39 ± 0.13	0.44 ± 0.16*,†††	13	0.58 ± 0.26***	49
90-Degree forward flexion while lifting 10 kg	0.55 ± 0.18	0.61 ± 0.21†††	11	0.81 ± 0.36***	47

* *p* < .05

** *p* < .01

*** *p* < .001 age-adjusted *p* values for each group compared with women with no deformities.

† *p* < .05

†† *p* < .01

††† *p* < .001 age-adjusted *p* values for those with grade 1 deformities compared with women with grade 2–3 deformities.

Compared with women with no vertebral deformity, those with deformities had lower mean bone density values (Table 1). The smallest difference was seen for LS aBMD, where the discrepancy between controls and those with moderate to severe deformities was just 7%. Excluding the 35 subjects with spine region aBMD obtained from a total-body scan (12 women with no deformity, 13 with grade 1 deformities only, and 10 with any grade 2–3 deformities) increased the discrepancy only slightly to 8%. The biggest difference was seen with LS trabecular vBMD, with similar changes evident in distal radius trabecular vBMD. By contrast, there were no significant differences in cortical vBMD in the radius. Differences across groups were less pronounced for bone geometry in the spine, with a tendency toward greater cross-sectional and endocortical areas in those with vertebral deformities but no differences in moment of inertia or section modulus (data not shown). However, compared with control women, apparent cortical thickness of the vertebra was significantly less in each deformity group.

For grade 2–3 but not grade 1 deformities, there also were reductions in microstructural variables by HRpQCT at the distal radius (Table 1). Thus BV/TV was 25% less among the women with moderate to severe deformities, with particular reductions in Tb.N and connectivity density. Conversely, Tb.Sp and Tb.Sp.SD tended to be greater, whereas the increase in SMI indicated a shift from platelike to more rodlike trabeculae. As observed in the spine, cortical thickness in the distal radius also was reduced among the women with moderate to severe deformities.

**Fig. 1:**
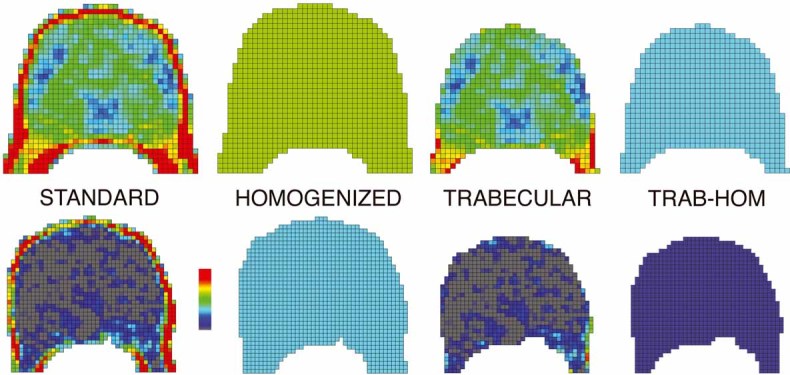
To illustrate the biomechanical effects of spatial variation in bone density, transverse cross sections of the finite-element model of a vertebra are shown for a subject with relatively strong bone (*top row*) and one with relatively weak bone (*bottom row*). Each finite element is assigned material properties based on vBMD data from the QCT scan for that element, ranging from high-density (*red*) to low-density (*gray*) bone. For each subject, cross sections are shown for four models: the unaltered vertebra (“STANDARD”); the vertebra with all elements assigned the average vBMD value for that model (“HOMOGENIZED”); a model consisting only of the trabecular compartment, in which the outer 2 mm of bone is virtually removed (“TRABECULAR”); and a homogenized version of that trabecular model with all elements assigned the average vBMD of the trabecular compartment (“TRAB-HOM”). In each case, the resulting finite-element models are virtually compressed to failure to estimate compressive strength, resulting in multiple strength outcomes for each subject.

Significantly reduced vertebral strength was observed in both deformity groups (Table 1). Overall FE vertebral compressive strength was 10% lower among women with grade 1 deformities compared with controls and 26% lower among those with grade 2–3 deformities. In the latter group, the strength of the “cortical” region (outer 2-mm layer of bone) was 84% that of controls, whereas trabecular compressive strength was only 68% as great. Consistent with this, the trabecular compartment accounted for 47% of overall vertebral strength, on average, compared with 52% among controls (*p* < .001). The deficit in trabecular compressive strength related about equally to reduced bone density and to heterogeneity in the spatial distribution of trabecular bone (Fig. 1). Thus bone strength in the trabecular compartment increased 1.4-fold when the subject-specific average vBMD was applied to all voxels (homogenized strength). Controls had 69% of the trabecular compressive strength that would be expected if they had fully homogeneous vertebra compared with 67% of expected strength among women with grade 1 deformities (*p* = .159). By contrast, women with moderate to severe deformities had only 58% of the strength that would be expected in the absence of trabecular heterogeneity (*p* < .001). In addition to axial loads, women with deformities also had lower vertebral stiffness under an AP bending moment (Table 1).

Women with deformities had significantly greater mean load-to-strength ratios than women with no deformities. The worst values of ϕ were observed for those with grade 2–3 deformities (Table 1). In each group, however, ϕ increased with the calculated load on the lumbar spine, from upright standing, to 45- and 90-degree forward bending, to bending 90 degrees at the waist while holding 10 kg. Using the latter load estimate, 22 women had ϕ > 1, the theoretical fracture threshold, and 91% of them had a deformity (22% of those with a severe deformity and 6% of those with grade 1 deformities alone). Conversely, only 2 of 90 control women had ϕ > 1.

By logistic regression, many bone density, structure, and strength variables were linked to an increased risk of vertebral deformities generally, but essentially all of them were more strongly associated with grade 2–3 vertebral deformities than with grade 1 deformities specifically (Table 2). For the bone density variables, *AUC*s ranged from 0.56 to 0.67 for discriminating women with any vertebral deformity from those with no deformities and from 0.61 to 0.79 for discriminating the moderate to severe deformities; in contrast, *AUC*s ranged just from 0.54 to 0.61 for discriminating grade 1 deformities from controls. Apparent cortical thickness was the most significant predictor of fracture risk among the vertebral bone geometry parameters, and a number of radius bone microstructure variables were associated with the moderate to severe deformities (Table 2). In particular, lower BV/TV, Tb.N, Tb.Th, and connectivity density were associated with increased vertebral fracture risk, whereas lower Tb.Sp and Tb.Sp.SD were protective, as was lower SMI (ie, more platelike than rodlike trabeculae). None of the associations between microstructure and grade 1 deformities were statistically significant. By contrast, most bone strength variables were associated with vertebral deformities of all types, although the ORs again were greatest for predicting moderate to severe deformities (Table 2). These results were unchanged by removal of the women on an osteoporosis-related drug (28 controls, 43 women with grade 1 deformities only, and 26 with any grade 2–3 deformities; data not shown).

**Table 2:** Relative Risk [Age-Adjusted Odds Ratios (ORs)] of Specific Vertebral Deformities (Semiquantitative Assessment) Among Postmenopausal Women by Five Main Predictor Variable Categories (Most Significant Predictor of Vertebral Fracture Within Each Category Is Indicated in Boldface Type)

	Any deformity		Mild (grade 1) deformity only		Any severe (grade 2–3) deformity	
			
Variable (units)	OR (95% CI)^a^	*AUC*	OR (95% CI)^a^	*AUC*	OR (95% CI)^a^	*AUC*
Bone density						
Lumbar spine (LS) aBMD (g/cm^2^)	1.3 (1.1–1.6)	0.58	1.2 (0.9–1.6)	0.55	1.8 (1.2–2.6)	0.64
Femoral neck aBMD (g/cm^2^)	1.5 (1.2–1.8)	0.60	1.3 (0.97–1.7)	0.56	2.2 (1.4–3.7)	0.69
Total radius aBMD (g/cm^2^)	1.4 (1.2–1.7)	0.59	1.2 (0.9–1.6)	0.54	2.0 (1.3–3.2)	0.67
LS total vBMD (mg/cm^3^)	1.8 (1.5–2.2)	0.66	**1.5 (1.1–2.0)**	**0.61**	3.5 (2.1–5.8)	0.78
LS trabecular vBMD (mg/cm^3^)	**1.9 (1.5–2.3)**	**0.67**	1.5 (1.1–2.0)	0.61	**4.0 (2.3–6.9)**	**0.79**
Distal radius trabecular vBMD (mg/cm^3^)	1.5 (1.2–1.8)	0.61	1.2 (0.9–1.6)	0.55	2.2 (1.4–3.4)	0.70
Distal radius cortical vBMD (mg/cm^3^)	1.2 (0.99–1.5)	0.56	1.2 (0.8–1.6)	0.55	1.4 (0.96–2.0)	0.61
Bone geometry (lumbar spine)						
Cross-sectional area (mm^2^)	0.9 (0.7–1.03)	0.55	0.8 (0.6–1.1)	0.54	0.7 (0.5–1.0)	0.57
Endocortical area (mm^2^)	0.8 (0.7–0.97)	0.57	0.8 (0.6–1.04)	0.56	0.6 (0.4–0.9)	0.61
Cortical area (mm^2^)	1.3 (1.04–1.6)	0.58	1.2 (0.9–1.6)	0.56	1.6 (1.1–2.3)	0.63
Apparent cortical thickness (mm)	**1.6 (1.3–1.9)**	**0.63**	**1.6 (1.2–2.1)**	**0.60**	**2.4 (1.6–3.8)**	**0.71**
Bone microstructure (distal radius*)*						
Trabecular bone volume/total volume (%)	**1.5 (1.2–1.8)**	**0.60**	1.2 (0.9–1.6)	0.55	**2.2 (1.4–3.3)**	**0.70**
Trabecular number (1/mm)	1.4 (1.1–1.7)	0.60	1.3 (0.9–1.7)	0.57	1.8 (1.2–2.8)	0.67
Trabecular thickness (µm)	1.3 (1.1–1.7)	0.57	1.0 (0.7–1.3)	0.51	1.8 (1.1–2.8)	0.65
Trabecular separation (Tb.Sp, µm)	0.8 (0.6–0.9)	0.58	0.9 (0.6–1.2)	0.55	0.6 (0.4–0.95)	0.64
Tb.Sp distribution (µm)	0.8 (0.6–0.95)	0.57	0.8 (0.6–1.2)	0.55	0.6 (0.4–0.9)	0.62
Connectivity density (1/mm^3^)	1.4 (1.2–1.8)	0.60	1.2 (0.9–1.6)	0.55	2.1 (1.3–3.3)	0.69
Structure model index	0.7 (0.5–0.8)	0.61	0.9 (0.7–1.2)	0.54	0.4 (0.2–0.7)	0.71
Cortical thickness (mm)	1.3 (1.1–1.6)	0.59	1.2 (0.9–1.6)	0.56	1.7 (1.1–2.6)	0.65
Bone strength (lumbar spine)						
Overall compressive strength (N)	1.7 (1.4–2.1)	0.64	**1.4 (1.1–1.8)**	**0.59**	2.9 (1.8–4.8)	0.74
Cortical strength (N)	1.5 (1.2–1.8)	0.61	1.3 (0.98–1.7)	0.57	2.1 (1.4–3.2)	0.69
Trabecular strength (N)	**1.8 (1.4–2.3)**	**0.65**	1.4 (1.1–1.8)	0.58	**3.7 (2.0–6.7)**	**0.76**
“Homogenized” trabecular strength (N)	1.7 (1.3–2.1)	0.63	1.4 (1.04–1.8)	0.58	2.7 (1.6–4.6)	0.73
Strength per unit density (N · cm^3^/mg)	1.5 (1.2–1.8)	0.60	1.1 (0.9–1.5)	0.52	2.1 (1.4–3.2)	0.70
AP bending stiffness (kN · m/rad)	1.4 (1.1–1.7)	0.59	1.2 (0.95–1.6)	0.55	1.9 (1.2–2.9)	0.66
Load-to-strength ratio (ϕ, lumbar spine)						
Upright standing	1.7 (1.4–2.1)	0.64	1.5 (1.1–2.2)	0.59	2.7 (1.7–4.4)	0.75
45-Degree forward flexion	1.7 (1.4–2.1)	0.65	**1.5 (1.1–2.2)**	**0.59**	2.7 (1.7–4.3)	0.75
90-Degree forward flexion	1.7 (1.4–2.1)	0.65	1.5 (1.1–2.2)	0.59	2.7 (1.7–4.3)	0.75
90-Degree forward flexion while lifting 10 kg	**1.8 (1.5–2.2)**	**0.65**	1.5 (1.1–2.2)	0.59	**2.9 (1.8–4.6)**	**0.75**

a Odds ratio per SD decrease (all variables except ϕ, which is per SD increase), adjusted for age.

## Discussion

The potential value of skeletal parameters for predicting vertebral fracture risk is underestimated by inadvertent inclusion of false-positive cases, but lack of concordance between various vertebral deformities and the symptoms that might signify true fracture(23) (24) precludes a clinical “gold standard.” Moreover, clinical (eg, semiquantitative) and quantitative (eg, morphometric) approaches are not highly correlated,(4) (25) and the problem is especially acute when assessing prevalent fractures where earlier measurements of deformity size or shape are not available. In particular, the weaker relation of mild vertebral deformities with structural damage, symptoms, or subsequent fractures suggests that some substantial fraction of them may represent false-positive results. In this study, postmenopausal women with moderate to severe (grade 2–3) vertebral deformities by the semiquantitative technique had much worse bone density, structure, and strength measurements compared with women with no deformities, but differences were less marked for women with only mild (grade 1) deformities. This is consistent with recent work showing that increasing vertebral fracture severity is associated with greater defects in bone microstructure, as assessed at the distal radius and iliac crest.(1) (2) However, the present analysis focuses specifically on the group with equivocal grade 1 deformities. They occupied an intermediate position between women with no evident deformity and those with definite vertebral fractures. The fact that they differed in many respects from control women with no deformity is contrary to our null hypothesis that prevalent grade 1 deformities, especially those found incidentally, merely represent extremes of the normal vertebral body shape distribution, earlier trauma, or juvenile vertebral epiphysitis (Scheuermann disease). Particularly noteworthy is the finding that various estimates of vertebral body strength were reduced among the women with grade 1 deformities compared with controls.

However, the observation that skeletal parameters were much less predictive of these grade 1 deformities than of the grade 2–3 deformities is also consistent with the suggestion that some mild deformities do not represent actual vertebral fractures.(4) In this regard, it is important to point out that no bone density, structure, or strength parameter was uniquely associated with the grade 1 deformities, which might thereby suggest a different pathogenetic process.(26) Rather, the associations were all similar in direction but weaker than those seen for grade 2–3 deformities. This emphasizes the need for continued efforts to better characterize the false-positive deformities identified by qualitative or quantitative morphometry.(25) Eliminating the dilution of effect from this source would increase the power of predictor variables substantially to discriminate women with mild deformities who are at greatest risk for progression to moderate to severe vertebral fractures.

These results support previous observations that disordered trabecular bone microstructure is important in fracture pathogenesis. For example, Sornay-Rendu and colleagues(1) found recently that all trabecular microstructural variables at the distal radius were significantly worse in women with vertebral fractures than in controls and that the deficits increased with the severity of the deformity; our results at the radius were similar. However, bone microstructure at one skeletal site may not correlate closely with another site,(27) so it was important to show directly in the weight-bearing spine that heterogeneity in trabecular vBMD is associated with a substantial reduction in vertebral failure load. Moreover, it is of interest that the homogenized trabecular strength (ie, after removal of intravertebral density variations) is independent of actual vBMD or aBMD values and therefore might represent a noninvasive assessment of trabecular bone quality in the spine. Indeed, when variability was virtually removed by assigning the subject-specific average vBMD in the trabecular compartment to each voxel, the estimated mean trabecular compressive strength increased from 1931 to 2853 N among the women with grade 2–3 deformities and from 2493 to 3466 N in those with only grade 1 deformities. Since ex vivo data document an adverse influence on vertebral strength of trabecular disruption, trabecular variation, and loss of trabecular number, it is not surprising that variability of bone density within the vertebral body is an important determinant of fracture susceptibility.(5) (16) (28)

Cortical bone is also important in vertebral fracture pathogenesis. Sornay-Rendu and colleagues(1) found that Ct.Th and cortical vBMD, but not cross-sectional area at the distal radius, were linked with the most severe vertebral deformities. Again, we found similar results except that the marginal association with distal radius cortical vBMD in our study was not statistically significant. However, the outer 2 mm of bone in the vertebral body, which approximates apparent cortical thickness by lumbar spine QCT,(11) carried 54% of the compressive load in controls, as found also in more detailed analyses of cadaver vertebrae.(29) Compared with controls, the strength of this “cortical” compartment was reduced by only 6% to 16% among the women with various vertebral deformities compared with the 12% to 32% reduction seen in trabecular compressive strength. Consequently, the “cortical” compartment bore nearly 61% of the load among women with grade 2–3 deformities, helping to explain associations of vertebral fracture risk with Ct.Th at the radius.(1)

Unfortunately, none of these cortical or trabecular parameters can be evaluated by DXA, which is, moreover, confounded by bone size. In addition, standard LS aBMD measurements include cortical bone in the posterior elements of the spine and other artifacts. Despite this, LS aBMD was associated (all *p* ≤ .001) with vertebral trabecular (*r* ^2^ = 0.59), cortical (*r* ^2^ = 0.71), and overall compressive strength (*r* ^2^ = 0.68), although each of them was a stronger predictor of vertebral fracture risk than LS aBMD in multivariate models. LS aBMD does predict vertebral fracture risk prospectively,(30) but the measure had only a moderate association with overall vertebral fracture risk in this study, albeit more case than control women were being treated with an antiresorptive agent. This seemed to have little effect on the parameter estimates, however, and the association with aBMD was not strengthened by adjustment for osteoporosis treatment or exclusion of treated women from the analysis. Likewise, whether or not the 35 women with spine regional aBMD assessed from a total-body scan were eliminated did not change the associations of LS or FN aBMD with fracture risk. Any overlap in aBMD is not due to inadvertent inclusion of fractured vertebrae (with their higher aBMD) in the scans because they were excluded according to ISCD guidelines, and FN aBMD performed only a little better. Some other studies have found similar results.(5) (31)

Our study had a number of strengths and limitations. The subjects were recruited from the community, but most of the women were white; men were excluded. The vertebral fracture cases all were confirmed by the widely accepted semiquantitative technique,(8) although reproducibility estimates were not obtained, and they included three times as many grade 1 deformities as studied previously.(1) Since cross-sectional assessment of vertebral fractures is problematic,(3) in the overall analysis we weighted the likelihood that different deformities represent actual vertebral fractures, thus acknowledging the equivocal nature of some grade 1 deformities.(4) Cases and controls were evaluated with state-of-the-art HRpQCT, which permits convenient assessment of trabecular microstructure, albeit at a distant site, the ultradistal radius. Both groups also were evaluated with central QCT of the spine, and we estimated vertebral strength from detailed FEA of the lumbar spine QCT images. Although the latter approach is restricted to the evaluation of isolated vertebral bodies, it provides excellent predictions of vertebral compressive strength compared with values obtained ex vivo.(15) Estimating the absolute value of the load component of the load-to-strength ratio is more problematic, however. This study employed lumbar spine loads associated with common activities of daily living, that is, bending at the waist. Bone loads associated with falls and more extreme activities of daily living likely would be greater, leading to more ϕ values > 1, but the actual loading event precipitating each vertebral fracture in this study was unknown, as typically is the case.

Using sensitive and relevant measures, this investigation confirms much recent work showing that numerous bone density, structure, and strength parameters predict vertebral fractures. All these parameters were much stronger predictors of moderate to severe vertebral deformities than of the mild vertebral deformities encountered more frequently in the population. This suggests that some grade 1 deformities represent false-positive cases. Conversely, the fact that their bone structure and strength parameters were significantly reduced compared with controls also indicates that many women in this group do have early osteoporotic fractures, so ignoring them completely may be inappropriate. Considerable progress in identifying vertebral fracture risk factors therefore could result from the development of more specific criteria for grade 1 deformities. If this continues to prove difficult,(3) however, skeletal assessment technologies will be needed that can better distinguish the true fractures.

## Disclosures

DMK has a financial interest in O.N. Diagnostics, and both he and the company may benefit from the results of this research. DK has equity interests in and is an employee of O.N. Diagnostics. In addition, RAR and JJC have a financial interest in the software used to analyze the computed tomographic scans. All the other authors state that they have no conflicts of interest.
